# Probiotics for preventing or treating COVID-19; a systematic review of research evidence and meta-analyses of efficacy for preventing death, severe disease, or disease progression

**DOI:** 10.12688/wellcomeopenres.18526.1

**Published:** 2022-12-05

**Authors:** Jawara Allen, Carlton A. Evans, Sumona Datta

**Affiliations:** 1 School of Medicine, Johns Hopkins University, Baltimore, Maryland, 21205, USA; 2 IFHAD: Innovation For Health and Development, Laboratory of Research and Development, Universidad Peruana Cayetano Heredia, Lima, 15102, Peru; 3 IFHAD: Innovation For Health and Development, Infectious Diseases and Immunity, Imperial College London and Wellcome Trust Imperial College Centre for Global Health Research, London, W12 0NN, UK; 4 Innovacion Por la Salud Y el Desarrollo (IPSYD), Asociación Benéfica Prisma, Lima, 15088, Peru; 5 Clinical Sciences, Liverpool School of Tropical Medicine, Liverpool, L3 5QA, UK

**Keywords:** Probiotics, COVID-19, SARS-CoV-2, Systematic Review, Meta-analysis

## Abstract

**Background:** COVID-19 variants threaten health globally. Despite improving vaccines and treatments, there is an urgent need for alternative strategies to prevent or reduce the severity of COVID-19. Potential strategies include probiotics, which are safe, inexpensive, globally available and have been studied previously in relation to respiratory infections.

**Methods:** We performed a systematic review and meta-analyses of experimental, trial or observational research evidence evaluating probiotics compared with control groups for preventing or treating COVID-19. We searched PubMed, ProQuest, Google Scholar and Web of Science bibliographic databases for studies published until December 6, 2021. We then performed meta-analyses for outcomes reported consistently across studies. Outcomes reported inconsistently or not amenable to meta-analysis were compared descriptively.

**Results:** We identified six eligible studies, which were all published in 2020 and 2021: one randomized controlled trial and five retrospective cohort studies. The only randomized controlled trial reported that groups that ingested probiotics compared with control groups that did not ingest probiotics did not differ significantly with respect to death, severe disease requiring admission to an intensive care unit or disease progression (all p>0.5). The five retrospective cohort studies reported various apparently beneficial and harmful COVID-19 outcome associations with probiotic ingestion. Meta-analyses revealed no significant associations between probiotic use and death, severe disease, or disease progression caused by COVID-19. Descriptive data revealed that probiotic ingestion was associated with a trend towards worsened duration of hospital stay, improvements in measures of respiratory condition and worsened disease duration. The evidence for these contradictory associations was weak because all studies were prone to bias and none were considered to be of high quality.

**Conclusions:** Current evidence does not suggest that probiotics affect COVID-19 severity or mortality. However, additional higher quality studies need to be conducted to definitively determine if probiotics would be a useful adjunctive treatment for COVID-19.

## Introduction

Since its emergence as a pathogen of pandemic potential in 2019, SARS-CoV-2 has spread rapidly globally causing high incidence and mortality of the predominantly respiratory disease COVID-19. With this rise has come an intense focus on finding agents that either prevent disease progression along the spectrum of mild to critical disease or that prevent death. Mild disease is characterized by signs/symptoms of COVID-19 but no shortness of breath, dyspnea or abnormal chest imaging ( Gandhi *et al.*, 2020). Moderate disease is characterized by signs of lower respiratory disease during clinical assessment or on imaging. Severe disease is characterized by oxygen saturation (SpO _2_) <94% on room air, a ratio of arterial partial pressure of oxygen to fraction of inspired oxygen (PaO _2_/FiO _2_) <300 mm Hg, a respiratory rate >30 breaths/min, or lung infiltrates >50%. Critical disease is characterized by respiratory failure, shock, or multiorgan dysfunction ( Gandhi *et al.*, 2020). So far, several therapies have been tested, but only a few have shown efficacy in the prevention of disease progression. These therapies include neutralizing monoclonal antibodies, systemic corticosteroids, IL-6 receptor blockers, and antivirals such as remdesivir; all of which , if available, need to be prescribed and monitored by licensed health care practitioners ( “Therapeutics and COVID-19: Living Guideline” n.d.). While these therapies have helped curb the impact of this viral infection, more affordable and accessible therapies are still needed. One potential treatment that is being actively pursued is modification of the gut microbiome *via* probiotics.

Probiotics are defined by the World Health Organization (WHO) as “live microorganisms that, when administered in adequate amounts, confer a health benefit on the host” ( Hill *et al.*, 2014). The route by which probiotics work to improve human health are thought to be associated with gut barrier reinforcement, normalization of perturbed microbiota, short chain fatty acid production, and immunological mechanisms including anti-inflammatory effects ( Hill *et al.*, 2014). Probiotics have long been studied for the prevention of diarrheal diseases ( Hempel *et al.*, 2012; Johnston *et al.*, 2012), but their use in prevention of respiratory infections has limited data. One systematic review found that probiotics were useful in preventing and reducing the duration of upper respiratory infections of all causes, but the evidence was of low quality ( Hao *et al.*, 2015). Other studies have shown that probiotics can reduce the incidence and severity of ventilator acquired pneumonia, which manifests as a lower respiratory infection ( Su *et al.*, 2020; Weng *et al.*, 2017). COVID-19 can be present as an upper respiratory infection in mild cases and a lower respiratory infection in moderate/ severe/ critical cases ( Walton *et al.*, 2021). The exact mechanism by which probiotics impact incidence and duration of respiratory tract infections has not been fully elucidated, but research suggests that alteration of the gut microbiome may reduce inflammation, particularly in older adults ( Vulevic *et al.*, 2015). This is important because individuals older than 65 years are at significantly increased risk of death due to COVID-19 ( Ho *et al.*, 2020).

While research progresses aiming to develop treatment and prevention measures that will help to end the COVID-19 pandemic, therapies to decrease the transmission, incidence, duration and severity of COVID-19 are needed to decrease challenges to healthcare systems and public health. Probiotics may help accomplish this, and anecdotal evidence has been published where probiotics have been provided to patients with COVID-19 ( Xu *et al.*, 2020). Probiotics have been shown to be safe in diverse settings, including some with vulnerable individuals, and thus can be tested and distributed quickly with little concern for major side effects ( Walton *et al.*, 2021). In fact, some countries have already suggested the consumption of specific probiotic species for nutritional value ( Smug *et al.*, 2014), and Italy has been regulating the use of probiotics in food since at least 2013 ( Hill *et al.*, 2014). Given the precedent for probiotic usage in many parts of the world, their potential use in the treatment of COVID-19 should be further investigated. Here in, we report a systematic review and meta-analyses of experimental, trial or observational research evidence evaluating probiotics compared with control groups for preventing or treating COVID-19.

## Methods

### Search strategy and information sources

This systematic review was conducted according to a protocol based on international standards that was developed before data collection commenced, and reported in accordance with the PRISMA guidelines ( Datta, 2022; Higgins *et al.*, 2021; Page *et al.*, 2021). The protocol was prepared prior to review commencement and shared between the authors but was not registered or published, however it can be requested from the authors.

We searched PubMed (RRID:SCR_004846), ProQuest (RRID:SCR_006093), Google Scholar (RRID:SCR_008878) and Web of Science (RRID:SCR_022706) using the following search terms: “probiotic” or “prebiotic” AND “COVID” or “COVID-19” or “SAR-COV-2”. Additionally, references cited by publications returned from these search criteria were hand-searched. The last date that each search was consulted was December 6, 2021.

### Inclusion criteria

For inclusion, full text, peer-reviewed articles in English or Spanish were considered. Additionally, articles included must have had a study design that contained a control group. These included case-control studies, cohort studies and randomized controlled trials. Studies must have been conducted in humans, and any dose, frequency or route of probiotics was acceptable. In terms of outcomes, studies were included that reported clinical outcomes directly related to prevention or treatment of human COVID-19, including disease or disease severity (such as rate of transfer to the intensive care unit (ICU) or need for mechanical ventilation, number of inpatient hospital days and death).

### Exclusion criteria

Studies that evaluated outcomes related to immune factors alone or non-respiratory symptoms alone were excluded. We also excluded studies in which participants were <18 years of age. Finally, we excluded review articles, statements or recommendations from professional bodies, or studies that were still ongoing.

### Selection process

JA and SD applied the exclusion criteria and used a pre-prepared form to extract the data from the included studies. Inclusion and exclusion criteria fulfilled by each study were tabulated to justify which studies continued into the next phase of the selection process. The summary of this process was presented in a flow diagram. JA and SD worked independently and extracted data simultaneously. Any discrepancies in selection or outcomes, described below, were discussed and resolved by JA, SD and CAE.

### Data items

The following data were extracted from each study if available: study design, month/year published, timeframe of study, location of study, COVID-19 treatment administered, probiotics used, how probiotics group was assigned, number of participants, median age of participants and percentage male representation.

The primary outcomes were extracted from each study and separated by probiotics treatment or control group and quantitatively compared between studies:

- (a) number of patients in group, number of patients who died;
- (b) number of patients transferred to the ICU or initiated on mechanical ventilation;
- (c) percentage of patients with moderate/severe COVID-19 *versus* mild COVID-19. In the studies that did not use presence of moderate/severe COVID-19 as an inclusion criterion, mild COVID-19 was either defined in the individual study or extracted using the number of patients not requiring oxygen support.

The following outcomes were also extracted but descriptively compared between groups as the definition of each outcome varied by study or the variable was not amenable to meta-analysis: number of patients whose respiratory disease progressed, disease duration, median number of inpatient days.

### Quality assessment

Both JA and SD assessed quality of the included studies using the Cochrane effective practice and organization of care (EPOC) risk of bias (RoB) tool ( Cochrane Effective Practice and Organisation of Care (EPOC), 2017). The results of the assessment were tabulated and are shown in the results.

### Data synthesis

Odds ratios with 95% confidence intervals (95% CI) were reported using available data extracted from each article for the primary outcomes: (a) death; (b) ICU admission/mechanical ventilation; and (c) progression to moderate-severe disease due to COVID-19. If the odds ratio was not reported, it was calculated using the numbers presented in each study. These quantitative data were presented both as tables and as forest plots. If there were at least two studies that evaluated the same primary outcome, then meta-analyses were performed with a random-effects model using the DerSimonian and Laird method. All analyses were carried out using Stata (RRID:SCR_012763) version 16 (STATA Corporation, College Station, TX, USA) using their official suite of meta-analysis commands (metan, metafunnel and metabias). As Stata is a proprietary software, similar analysis could be carried out using the freely available ReviewManager (RevMan, RRID:SCR_003581).

All non-primary outcomes that were assessed in the studies are presented descriptively.

### Heterogeneity and bias evaluation

Heterogeneity was assessed visually by forest plots, and analytically by I ^2^ and Cochrane Q test. However due to the *a priori* assumption of limited data there were no plans for sub-group analyses nor meta-regression analysis. If there was significant heterogeneity in any meta-analysis, the cause was investigated and a sensitivity analysis without the potential contributing studies was considered if there was sufficient studies.

Publication bias was determined by visualization of funnel plots and Egger test for each primary outcome. Egger testing could only be performed if there were at least three studies in the analysis.

## Results

### Study characteristics

**Figure 1.**
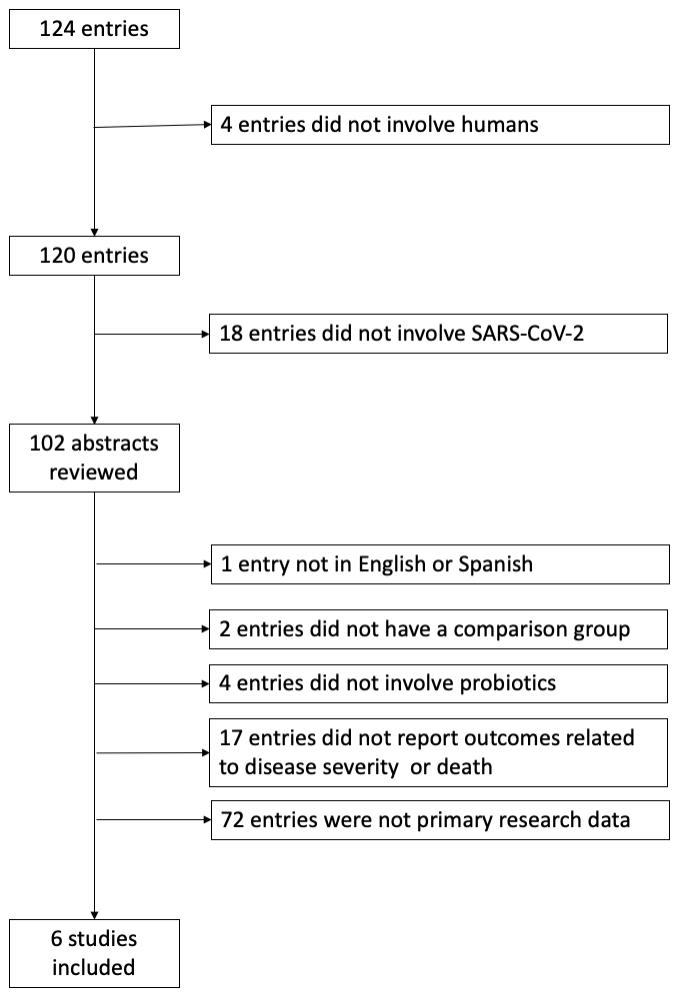
Study Selection.

Our search strategy identified 124 studies, six of which fulfilled our eligibility criteria. Reasons for exclusion of each article are shown in the flow diagram ( Figure 1). One of six articles was published in 2020 and the other five were published in 2021. Of the six eligible studies, one was a randomized controlled trial, and five were retrospective cohort studies. The studies were each conducted in different countries found in Africa, Asia or Europe. All the studies only included data for patients with a confirmed diagnosis of COVID-19 using real time polymerase chain reaction (PCR) on oropharyngeal or nasopharyngeal samples, although at least one study ( Hegazy *et al.*, 2022) included participants who had been taking probiotics since prior to their COVID-19 diagnosis. COVID-19 treatment varied widely by study, likely due to the rapidly changing international guidelines around treatment of COVID-19 in 2020. As shown in Table 1, four of the six eligible studies utilized hydroxychloroquine or chloroquine phosphate in their treatment regimen; four of the six eligible studies utilized an anti-interleukin six monoclonal antibody therapy; four of the six eligible studies utilized antiviral therapies; and two of the six eligible studies utilized corticosteroids as therapy. Women accounted for at least 40% of subjects in all studies, and the median age of study participants was > 55 years old.

**Table 1.** Study Characteristics. Note that N/A=not applicable and IQR=interquartile range.

Citation	Study Design	Month/ Year published	Timeframe of study	Location	(Hydroxy) Chloroquine used as treatment?	Anti-IL6 Ab used as treatment?	Antivirals used as treatment?	Corticosteroids used as treatment?	Probiotics given as treatment?	Probiotics used	How was probiotic treatment decided?	# of participants	Median age (IQR)	% males overall
Hegazy *et al.*, 2022	Retrospective cohort study	06/21	05/20 - 07/20	Cairo, Egypt	Yes	No	No	No	No	Probiotic yogurt (PY)	N/A	200	Not Reported	Not Reported
d'Ettorre *et al.*, 2020	Retrospective cohort study	07/20	03/9/20 - 04/4/20	Rome, Italy	Yes	Yes	No	No	Yes	Sivomixx® a multi-strain product containing five strains of *Lactobacilli,* two strains of *Bifidobacteria*, and one strain of *Streptococcus thermophilus*	Unclear	70	59 (50,70)	59% male
Ivashkin *et al.*, 2021	Randomized, controlled trial	10/21	10/20 - 03/21	Moscow, Russia	No	Yes	Yes	Yes	Yes	L *. rhamnosus, B.* * bifidum, B. longum subsp. infantis , B.* * longum subsp. longum*	Random	200	64 (54-70) *	46% male
Ceccarelli *et al.*, 2021	Retrospective cohort study	01/21	03/6/20 - 04/26/20	Rome, Italy	Yes	Yes	Yes	No	Yes	Sivomixx® a multi-strain product containing five strains of L *actobacilli*, two strains of *Bifidobacteria,* and one strain of *Streptococcus thermophilus*	Presence of intestinal symptoms	200	63 (54, 75)	57% male
Li *et al.*, 2021	Retrospective cohort study	06/21	02/3/20 - 02/20/20	Wuhan, China	Yes	No	Yes	Yes	Yes	Several probiotic regimens were used: 1.) *B. infantis,* * L. acidophilus,* Dung enterococcus, *B. cereus 2.) * *B. longum, L. bulgaricus, S. * *thermophiles 3.) E. faecium, * *B. subtilis*	Physician discretions	311	60 (12) **	48% male
Bozkurt & Bilen, 2021	Retrospective cohort study	11/21	11/1/20 - 12/15/20	Istanbul, Turkey	No	Yes	Yes	No	Yes	*B. animalis sp. Lactis BB-12* strain	Denied/Did not tolerate standard COVID-19 treatment	44	Not Reported	Not Reported

* Median age reported by group: 65 (59-71) for probiotics group and 64 (54-70) for control group

** Reported as mean (SD)

### Probiotics provided

With regards to the probiotics used, five of the six eligible studies specified the probiotic strains used. Of those, three of the five studies utilized a combination of *Lactobacillus* species, *Bifidobacterium* species, and *Streptococcus thermophilus*, one of the five studies utilized a combination of *Lactobacillus* species and *Bifidobacterium* species, and one of the five studies utilized *Bifidobacterium* species alone. The exact bacterial species utilized are detailed in Table 1. All five studies that utilized a specific combination of probiotic species were performed in the inpatient setting.

### Outcomes

No eligible studies were identified that assessed associations between probiotic use and prevention of COVID-19 diagnosis and/or transmission.

Eligible studies were identified that were suitable for data synthesis by meta-analysis concerning associations between probiotic consumption and COVID-19 causing: (A) death; (B) need for mechanical ventilation or ICU admission; and (C) moderate/severe disease progression. Eligible studies were identified that were suitable for only descriptive data synthesis ( *i.e.*, not suitable for meta-analysis) concerning associations between probiotic consumption and COVID-19 affecting: (D) hospital inpatient stay; (E) worsening respiratory condition; and (F) disease duration. These are detailed immediately below.

### Data synthesis of study outcomes by meta-analyses

**Figure 2.**
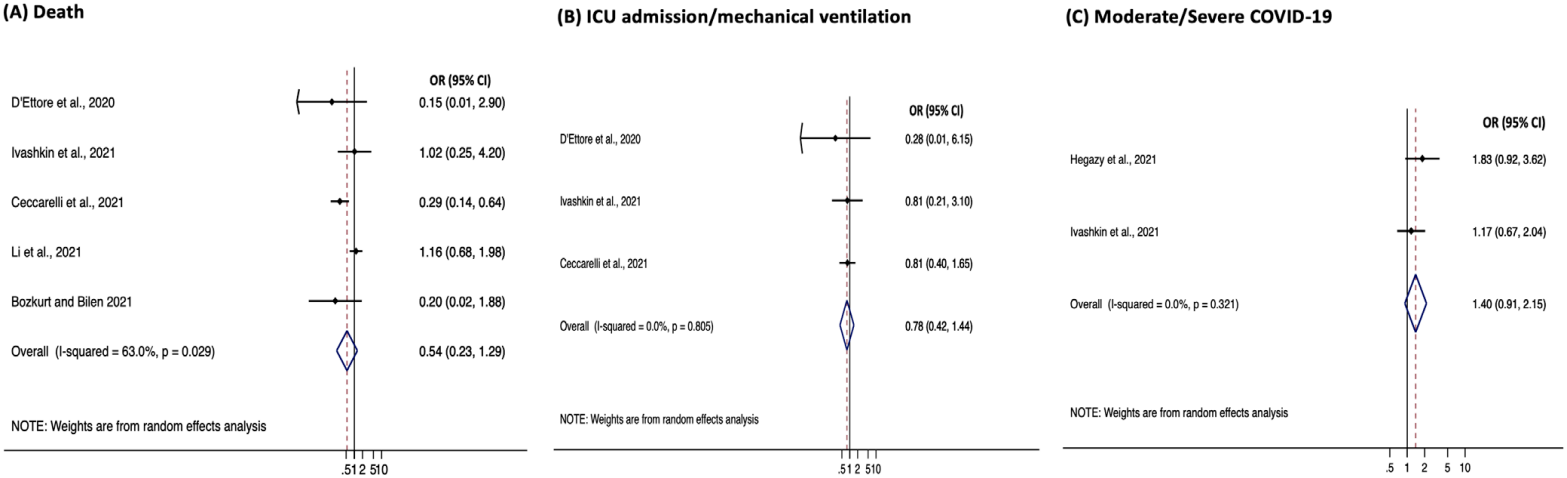
Meta-analyses forest plots. Plots for ( **A**) death, ( **B**) ICU admission/mechanical ventilation and ( **C**) moderate/severe COVID-19. Note that OR=odds ratio, 95%CI=95% confidence intervals and ICU=intensive care unit.

*(A) Death from COVID-19.* There were five studies that reported death from COVID-19 as a study outcome, and these five studies included data from 825 patients. The proportion of patients who died during the study ranged from 4% to 30% in the control groups (N=467) and 0% to 11% in the probiotics groups (N=358, Table 2). Only one study, which was a retrospective cohort study, reported a statistically significant association between the number of patients who died from COVID-19 and exposure to probiotics (OR 0.29, 95% CI 0.14, 0.64) ( Ceccarelli *et al.*, 2021). Meta-analysis revealed a non-significant pooled odds ratio of 0.54 (95% CI 0.23, 1.28, p=0.3) for probiotics use to prevent death due to COVID-19, with significant heterogeneity between studies (I ^2^=63%, p=0.03, Figure 2). There was a lot of variability with regards to study design, the COVID-19 treatment, probiotics used and to whom they were provided ( Table 1). The number of deaths in the study by Li *et al.* was unclear and an OR was not reported ( Table 2), thus we extracted data using the most likely interpretation of statements made within their published manuscript ( Li *et al.*, 2021).

**Table 2.** Reported Study Outcomes. Note N/A= not applicable, ICU=intensive care unit, IQR=interquartile range, OR=odds ratio, 95%CI= 95% confidence intervals.

Citation	Control					Probiotics					Deaths OR (95% CI)	ICU/ Mechanical Ventilation OR (95% CI)	Moderate/ Severe COVID-19 OR (95% CI)	Inpatient days Delta (reported P-value)
	# of participants	% Death	% ICU/ Mechanical Ventilation	% Moderate/ Severe COVID-19	Days inpatient median (IQR)	# of participants	% Death	% ICU/ Mechanical Ventilation	% Moderate/ Severe COVID-19	Days inpatient median (IQR)				
Hegazy *et al.*, 2022	52	N/A **	N/A **	29.00%	N/A	148	N/ **	N/A **	43.00%	N/A	N/A	N/A	1.83 (0.92, 3.62)	N/A
d'Ettorre *et al.*, 2020	42	10%	5.0%	N/A *	Not Reported	28	0%	0%	N/A *	Not Reported	0.15 (0.008,2.9)	0.28 (0.01, 6.1)	N/A *	N/A
Ivashkin *et al.*, 2021	101	4%	5.0%	44.00%	11 (9, 14)	99	4%	4.00%	47.00%	11 (10, 14)	1.02 (0.25,4.02)	0.81 (0.21, 3.1)	1.17 (0.67, 2.04)	0 (0.4)
Ceccarelli *et al.*, 2021	112	30%	21%	N/A *	14 (8, 23)	88	11%	18%	N/A *	20 (11, 31)	0.29 (0.14,0.64)	0.81 (0.4, 1.65)	N/A *	6 (0.01)
Li *et al.*, 2021	188	22%	Not Reported	N/A *	20 (Not Reported)	123	24%	Not Reported	N/A *	32 (Not Reported)	N/A	N/A	N/A *	12 (<0.001)
Bozkurt & Bilen, 2021	24	21%	Not Reported	N/A *	13.6 (Not Reported)	20	5%	Not Reported	N/A *	7.6 (Not Reported)	0.2 (0.02,1.88)	N/A	N/A *	-6 (<0.001)

* All patients had moderate/severe COVID-19 upon admission, as a part of the inclusion criteria

** Patients with severe COVID-19 were excluded from the study

*(B) Need for mechanical ventilation or ICU admission due to COVID-19.* There were three studies that reported the need for mechanical ventilation or ICU admission as a study outcome. These three studies included data from 470 patients in which the proportion of patients who required mechanical ventilation or ICU admission ranged from 5% to 21% of patients (N=255) in the control groups and 0% to 18% of patients (N=215) in the probiotics groups ( Table 2). Meta-analysis demonstrated no statistically significant associations (OR=0.78, 95%CI=0.42, 1.44, p=0.4) ( Figure 2) with no heterogeneity between studies (I ^2^=0.0%, p=0.8).

*(C) Moderate/severe disease progression due to COVID-19.* The percentage of patients with moderate/severe COVID-19 was reported in all studies, but only two studies made a comparison with the percentage of patients with mild COVID-19 who were also provided probiotics (N=400). In neither study was there a statistically significant association observed between probiotics use and COVID-19 severity ( Table 2). The lack of association was also demonstrated in the meta-analysis (OR=1.4, 95% CI=0.91, 2.2, p=0.1) ( Figure 2). In both eligible studies and in the meta-analysis there was a non-significant trend towards probiotic therapy having an adverse effect, potentially increasing progression to moderate/severe COVID-19. No heterogeneity was observed between the two studies (I ^2^=0.0%, p=0.3).

### Descriptive data of other study outcomes

Due to inconsistent reporting across studies, including inconsistencies in the units used to report variables, the following study outcomes could only be described per study and not summarized in a meta-analysis.

*(D) Hospital inpatient stay.* The median number of inpatient days was reported by four studies with a range of 11 to 20 days in the control groups and 7.6 to 32 days in the probiotics groups ( Table 2). Three of four of these studies reported a significant difference in inpatient days between control and probiotics groups. Of these, two studies reported that patients receiving probiotics were more likely to have a *longer* inpatient stay than control patients ( d’Ettorre *et al.*, 2020; Li *et al.*, 2021), while one study reported that patients receiving probiotics were more likely to have a *shorter* inpatient stay than control patients ( Bozkurt & Bilen, 2021). We therefore conclude that the eligible studies suggested no consistent association between probiotic administration and duration of hospital stay for COVID-19, with a trend towards probiotics worsening duration of hospital stay.

*(E) Worsening respiratory condition.* Two studies reported the number of patients whose respiratory condition worsened, but the criteria they used to define that worsening varied. One retrospective cohort study used a general linear mixed model and showed an 8-fold (OR=8.62,95% CI= 1.65, 44.98, p=0.01) decreased risk of respiratory failure in those with severe COVID-19 disease (defined as the need for prone ventilation or extracorporeal membrane oxygenation) in patients administered probiotics ( d’Ettorre *et al.*, 2020). Another retrospective cohort study reported a statistically significant improvement in the percentage of patients found to have resolution of thoracic CT findings 3 weeks after COVID-19 diagnosis in the probiotics group when compared to the control group (p < 0.01) (no odds ratio reported) ( Bozkurt & Bilen, 2021). We therefore conclude that two of the eligible studies reported associations between probiotic administration and improvements in measures of respiratory condition due to COVID-19.

*(F) Disease duration.* Two studies attempted to assess disease duration. One study found no association between probiotic usage and total duration of disease ( Ivashkin *et al.*, 2021), while the other found that individuals given probiotics had a statistically significantly increased virus clearance time (P < 0.001) *i.e.*, probiotic administration worsened this measure of COVID-19 severity ( Li *et al.*, 2021). We therefore conclude that two of the eligible studies assessed disease duration and one of these provided evidence suggesting that probiotics worsened disease duration.

### Risk of bias

**Figure 3.**
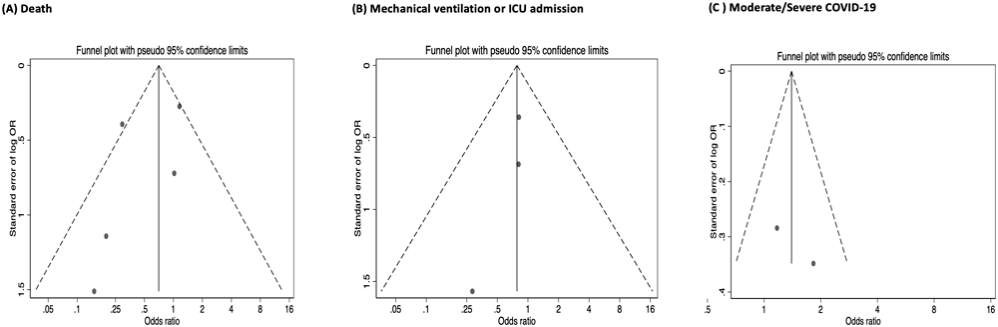
Funnel plot to assess missing data bias. Plots for ( **A**) death, ( **B**) ICU admission/mechanical ventilation and ( **C**) moderate/severe COVID-19. Note that OR=odds ratio and ICU=intensive care unit.

Notably, all of the studies, except the one randomized control trial, reported statistically significant differences between control and probiotics groups. Funnel plots ( Figure 3) suggest asymmetry in published OR for all the primary outcomes, thus demonstrating missing data bias although Egger testing showed no small study effect (all p>0.2).

### Study quality

Potential bias, as identified by the Cochrane Review EPOC RoB tool, was present for all the retrospective cohort studies analyzed ( Table 3). The main sources of bias were differences between baseline characteristics, selective reporting with unclear description of how outcomes were defined, and other biases associated with study design or description. These differences in baseline characteristics could be related to how treatment groups were allocated, and consequently allocation was likely to be associated with disease severity or symptom presentation at the time of hospitalization. The only randomized control trial included in this review was considered to be of intermediate quality because the method of allocation concealment was not detailed in the manuscript.

**Table 3.** Quality of assessment using theCochrane effective practice and organization of care (EPOC) risk of bias (RoB) tool.

Citation	Incomplete outcome data assessed	Free of selective reporting	Free from contamination	Baseline characteristics similar	Baseline outcomes similar	Free of other bias	Quality
*Hegazy et al., 2022*	Low Risk	Low Risk	High Risk	Unclear Risk	Unclear Risk	High Risk	*Poor*
*d'Ettorre et al., 2020*	Low Risk	High Risk	Low Risk	Low Risk	High Risk	High Risk	*Poor*
*Ivashkin et al., 2021*	Low Risk	Low Risk	Low Risk	Low Risk	Low Risk	Low Risk	*Intermediate*
*Ceccarelli et al., 2021*	Low Risk	Low Risk	Low Risk	High Risk	Low Risk	Low Risk	*Poor*
*Li et al., 2021*	Low Risk	High Risk	High Risk	High Risk	Low Risk	Low Risk	*Poor*
*Bozkurt & Bilen, 2021*	Low Risk	Low Risk	Low Risk	Unclear Risk	Low Risk	High Risk	*Poor*

## Discussion

We present a systematic review of controlled research evidence evaluating probiotics for preventing or treating COVID-19. The available publications allowed us to perform meta-analyses of probiotic efficacy for preventing death, severe disease, or disease progression and descriptive data synthesis of evidence that probiotics may influence hospital inpatient stay, worsening respiratory condition and disease duration. We identified no eligible studies evaluating probiotics for preventing COVID-19 diagnosis or transmission. The six eligible studies all assessed reduction of COVID-19 death or disease severity; five were retrospective cohort studies that were classified as having poor quality according to the EPOC RoB tool ( Higgins *et al.*, 2021) and there was one randomized controlled trial. Neither the meta-analyses not the descriptive data synthesis provided consistent evidence that probiotics affect the COVID-19 mortality, disease severity or duration.

Probiotic consumption was associated with significantly longer hospital stays in two out of four studies that assessed this outcome, contrasting with probiotic therapy being associated with a significantly shorter hospital stay due to COVID-19 in one study, suggesting a possible trend consistent with probiotics worsening rather than shortening COVID-19 duration. In both eligible studies that assessed the progression of COVID-19 respiratory disease, probiotics were associated with improvements. However, both studies were retrospective cohort studies that scored as having high risks for several forms of bias. Specifically, in one study, how baseline characteristics were factored into their mathematical model of progression to respiratory failure was not delineated ( d’Ettorre *et al.*, 2020), and in the other, the blinding of radiologists to the treatment allocation was not reported, baseline characteristics between probiotic and control groups were not detailed, and assignment to the probiotic group was based on inability to tolerate, or lack of desire to take, standard COVID-19 treatment ( Bozkurt & Bilen, 2021). Moreover, the one eligible study that was a randomized controlled trial reported no difference in progression to respiratory failure (as defined by need for mechanical ventilation) ( Ivashkin *et al.*, 2021), calling into question the potential association between probiotic use and progression of respiratory disease found in the retrospective cohort studies that had high risk of bias.

Overall, the conclusions of this systematic review and meta-analyses were limited by the quality of the studies assessed. All the retrospective cohort studies scored as having high risk for at least one form of bias and many scored high risk for multiple forms of bias ( Table 3). The poorly defined disease outcomes, heterogeneity of outcomes reporting and suboptimal study design in several studies made comparison across studies and validation of their findings challenging. Furthermore, the differences in allocation of probiotics, type of probiotics, and treatment strategies between the studies adds to the uncertainty in the pooled estimates.

Several other reviews on this topic have been published within the last year. Many of the previous reviews included trials or studies that were ongoing as most had been initiated in 2020 ( Hawryłkowicz *et al.*, 2021; Kurian *et al.*, 2021; Nayebi *et al.*, 2022; Peng *et al.*, 2021; Spagnolello *et al.*, 2021). As a result, our systematic review adds to the current literature by analyzing the outcomes from trials that were not complete at the time of previous reviews. Additionally, most previous reviews have focused on outcomes associated with disease severity such as factors involved in the inflammatory immune response. While these factors are important and have been shown to be associated with disease outcomes ( Lee *et al.*, 2021; Stoeckle *et al.*, 2022), we elected to focus on direct clinical outcomes in order to establish a more robust connection between probiotics and COVID-19 outcomes. Notably, there were no studies that were identified by our search strategy that investigated the prevention of COVID-19 in the community nor effects of COVID-19 vaccine response with probiotic use, which is an obvious gap in research that should be addressed.

Most studies assessed here used a combination of *Lactobacillus* and *Bifidobacterium species* in their probiotic treatment regimens. *Bifidobacterium* and *Lactobacillus* have been studied extensively and are frequently found in yogurts and other dairy products ( Zhang *et al.*, 2018). Thus, they are frequently used in trials ( Yan & Polk, 2011). Certain strains of *Bifidobacterium* have been shown to exert anti-inflammatory and immunomodulatory effects ( Bozkurt & Quigley, 2020) and strains of *Lactobacillus* have been shown to augment both the innate and adaptive immune responses ( Darbandi *et al.*, 2021). Additionally, both genera of bacteria have been shown to have a beneficial impact on the progression of other respiratory infections in several clinical trials ( Darbandi *et al.*, 2021). *Streptococcus thermophilus* was also used in several of the studies analyzed, but evidence supporting its use as a beneficial probiotic in respiratory infections is more limited. The probiotics used in future trials should be standardized to allow for comparison across studies. Because of their extensive use in prior research and their proven safety profiles, we propose that *Lactobacillus and Bifidobacterium species* should be considered as a part of this standardization.

As the COVID-19 pandemic continues transitioning from a new, emerging disease to an endemic illness causing sustained challenges to healthcare systems and public health, there is an urgent need to investigate inexpensive, accessible treatment and prevention strategies, particularly ones that can be utilized in community, outpatient setting. The eligible studies identified in this review only focused on treatment with adjunctive probiotics for patients with confirmed COVID-19 and did not support its use to prevent death, severe or prolonged disease caused by COVID-19. However due to the poor quality of the studies, more robust randomized controlled trials or larger retrospective cohort studies are needed and updated meta-analyses will be required including future evidence and SARS-CoV-2 variants.

## Data Availability

All data underlying the results are available as part of the article and no additional source data are required. Harvard Dataverse: PRISMA checklist for ‘Probiotics for preventing or treating COVID-19; a systematic review of research evidence and meta-analyses of efficacy for preventing death, severe disease, or disease progression’. https://doi.org/10.7910/DVN/1MGEMK ( Datta, 2022). Data are available under the terms of the Creative Commons Zero "No rights reserved" data waiver (CC0 1.0 Public domain dedication).
