# Supplementary material for: Probiotics for preventing or treating COVID-19; a systematic review of research evidence and meta-analyses of efficacy for preventing death, severe disease, or disease progression
Source: Wellcome Open Res. 2022 Dec 5;7:292. [Version 1] doi: 10.12688/wellcomeopenres.18526.1 (PMC11447443; doi:10.12688/wellcomeopenres.18526.1)
Supplement: Peer review history

# Reviewer report: Reviewer response for version 1

**DOI:** 10.21956/wellcomeopenres.20544.r60175

**Published:** 2024-10-02

**Authors:** Janet Schloss

**Affiliations:** 1 National Centre for Natural Medicine (NCNM), Faculty of Health, Southern Cross University, Lismore, Australia

This manuscript describes a systematic review and meta-analysis of studies on probiotics and COVID-19. The methods are well set out and reproducible. The writing and evaluation were of a high standard and very balanced in view. The discussion was well addressed with the conclusions meeting the findings. 

 I would recommend to index as is.

Are the rationale for, and objectives of, the Systematic Review clearly stated?

Yes

Is the statistical analysis and its interpretation appropriate?

Yes

Are sufficient details of the methods and analysis provided to allow replication by others?

Yes

Are the conclusions drawn adequately supported by the results presented in the review?

Yes

Reviewer Expertise:

Health, nutrition, nutraceuticals, cannabis

I confirm that I have read this submission and believe that I have an appropriate level of expertise to confirm that it is of an acceptable scientific standard.

# Reviewer report: Reviewer response for version 1

**DOI:** 10.21956/wellcomeopenres.20544.r62798

**Published:** 2024-10-02

**Authors:** Ioannis Alexandros Charitos

**Affiliations:** 1 Department of Emergency and Urgency, National Poisoning Center, Riuniti University Hospital of Foggia, Riuniti University Hospital of Foggia & University of Bari, Foggia, Italy

The research article has good scientific impact. It would be improved with the following changes:

1. A scheme/figure is needed to describe the inclusion and exclusion criteria.
2. There is no summary table with the dosages used, the combinations of probiotic strains, or the duration of the treatment and patient outcome. This is useful for the clinician who wants to understand how they might use them.
3. Please make the conclusions clearer, creating a percentage figure (how many articles were in favour and how many were not or other).

Are the rationale for, and objectives of, the Systematic Review clearly stated?

Yes

Is the statistical analysis and its interpretation appropriate?

Yes

Are sufficient details of the methods and analysis provided to allow replication by others?

No

Are the conclusions drawn adequately supported by the results presented in the review?

Yes

Reviewer Expertise:

Microbiota, Sepsis, Biotoxins, Intensive care

I confirm that I have read this submission and believe that I have an appropriate level of expertise to confirm that it is of an acceptable scientific standard.
